# Supplementary material for: Strategies adopted by men to deal with uncertainty and anxiety when following an active surveillance/monitoring protocol for localised prostate cancer and implications for care: a longitudinal qualitative study embedded within the ProtecT trial
Source: BMJ Open. 2020 Sep 9;10(9):e036024. doi: 10.1136/bmjopen-2019-036024 (PMC7482454; doi:10.1136/bmjopen-2019-036024)
Supplement: Supplementary data [file bmjopen-2019-036024supp001.pdf]

Appendix A Topics in interview guide

- Decision-making whether to participate in the ProtecT study
- Experiences of the diagnostic process
- Impact of prostate cancer diagnosis at time of diagnosis and ongoing
- Decision-making whether to accept random allocation to a treatment or to choose a treatment
- Views and experiences of AM and changes over time
- Views on radical treatments
- Decision-making whether to initiate radical treatment (if applicable)
- Experiences of radical treatment and its sequelae (if applicable)
